# Electron-phonon interaction and pairing mechanism in superconducting Ca-intercalated bilayer graphene

**DOI:** 10.1038/srep21414

**Published:** 2016-02-19

**Authors:** E. R. Margine, Henry Lambert, Feliciano Giustino

**Affiliations:** 1 Department of Physics, Applied Physics and Astronomy, Binghamton University, State University of New York, PO Box 6000, Binghamton, New York 13902-6000, USA; 2 Department of Materials, University of Oxford, Parks Road, Oxford OX1 3PH, United Kingdom

## Abstract

Using the *ab initio* anisotropic Eliashberg theory including Coulomb interactions, we investigate the electron-phonon interaction and the pairing mechanism in the recently-reported superconducting Ca-intercalated bilayer graphene. We find that C_6_CaC_6_ can support phonon-mediated superconductivity with a critical temperature *T*_*c*_ = 6.8–8.1 K, in good agreement with experimental data. Our calculations indicate that the low-energy Ca_*xy*_ vibrations are critical to the pairing, and that it should be possible to resolve two distinct superconducting gaps on the electron and hole Fermi surface pockets.

Recent progress in the fabrication of metal-intercalated bilayer graphene^1^,^2^,^3^, the thinnest limit of graphite intercalation compounds (GICs), opened promising new avenues for studying exotic quantum phenomena in two dimensions. Recently two independent studies reported the observation of superconductivity in Ca-intercalated bilayer graphene at 4 K^4^ and in Ca-intercalated graphene laminates around 6.4 K^5^, while a third study presented evidence for superconductivity in Li-decorated monolayer graphene around 5.9 K^6^. The electron-phonon interaction is expected to play a central role in these observations, hence it is important to develop a detailed understanding of electron-phonon physics in these newly-discovered superconductors.

The strength of the electron-phonon coupling (EPC) in graphite and graphene has been widely investigated using angle-resolved photoelectron spectroscopy (ARPES), however the interpretation of the results is not always straightforward^7^,^8^,^9^,^10^,^11^,^12^,^13^. For example the anisotropy of the EPC in alkali-metal decorated graphene generated significant debate in relation to the role of van Hove singularities^10^,^11^,^12^,^14^. Furthermore the relative importance of the π^*^ bands and of the interlayer (IL) band in the pairing mechanism of bulk CaC_6_^15^ has been the subject of an intense debate. Indeed several studies suggested that superconductivity arises from the EPC of both bands^13^,^16^,^17^,^18^,^19^,^20^, while ARPES studies proposed that superconductivity sets in either the π^*^ bands or the IL band alone^21^,^22^. This debate was re-ignited by the experimental observation of a free-electron IL band in Ca- and Rb-intercalated bilayer graphene^1^,^2^,^3^. The possibility of superconductivity in C_6_CaC_6_ was suggested theoretically based on the analogy with bulk CaC_6_^23^,^24^, however predictive *ab initio* calculations of the critical temperature have not yet been reported.

In this work we elucidate the role of the electron-phonon interaction in the normal and in the superconducting state of C_6_CaC_6_ by performing state-of-the-art *ab initio* calculations powered by electron-phonon Wannier interpolation^25^,^26^. For the normal state we study the electron self-energy and spectral function in the Migdal approximation; for the superconducting state we solve the anisotropic Migdal-Eliashberg equations including Coulomb interactions from first principles. Our main findings are: (i) superconductivity in C_6_CaC_6_ can be explained by a phonon-mediated pairing mechanism; (ii) in contrast to bulk CaC_6_, low-energy Ca vibrations are responsible for the majority of the EPC in the superconducting state; (iii) unlike bulk CaC_6_, C_6_CaC_6_ should exhibit two superconducting gaps. For clarity all technical details are described in the Methods.

Figure 1(a) shows a ball-and-stick model of Ca-intercalated bilayer graphene, while Fig. 1(b–d) show the corresponding band structure, Brillouin zone, and Fermi surface, respectively. Two sets of bands cross the Fermi level around the Γ point. The bands labeled as α^*^ and β^*^ in Fig. 1(b) represent π^*^ states, and are obtained by folding the Dirac cone of graphene from K to Γ, following the superstructure induced by Ca. The band labeled as IL is the Ca-derived nearly-free electron band, which disperses upwards from about 0.5 eV below the Fermi energy at Γ^1^,^23^,^24^. In Fig. 1(d) we see two sets of Fermi surface sheets: triangular hole pockets around the K′ points, corresponding to the 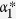 states; and a bundle of small electron pockets around Γ, which arise from the other π^*^ states (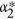, 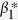, and 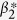) and from the IL band.

In order to quantify the strength of EPCs for each of these bands we calculate the spectral function in the normal state using the Migdal approximation^27^. Figure 1(e–g) show that the EPC induces sudden changes of slope in the bands, which are referred to as ‘kinks’ in high-resolution ARPES experiments. A pronounced kink is seen in all bands at a binding energy of 180 meV. This feature can be assigned to the coupling with the in-plane C_*xy*_ stretching modes^28^ (see Supplementary Fig. S1 for the phonon dispersion relations). The occurrence of this high-energy kink in the π^*^ bands is consistent with the observed broadening of the quasiparticle peaks in the ARPES spectra of bulk CaC_6_ in the same energy range^13^. This feature results from a sharp peak at 180 meV in the real part of the electron self-energy of the π^*^ electron pockets, as shown in Supplementary Fig. S2. A second kink is clearly seen at a binding energy of 70 meV, and is mostly visible for the π^*^ bands defining the hole pockets [magenta line in Fig. 1(e)] and for the interlayer band [cyan line in Fig. 1(g)]. This low-energy kink corresponds to a second, smaller peak at the same energy in the real part of the electron self-energy (Supplementary Fig. S2), and arises from the coupling with the out-of-plane C_*z*_ modes of the graphene sheets. Closer inspection reveals also a third kink around 12 meV, however this feature is hardly discernible and is unlikely to be observed in ARPES experiments. This faint structure arises from the coupling to the in-plane Ca_*xy*_ vibrations, and can be seen more clearly in the real and the imaginary parts of the electron self-energy in Supplementary Fig. S2.

From the calculated electron self-energy we can extract the electron-phonon mass enhancement parameter *λ*_F_ for each band using the ratio between the bare and the renormalized Fermi velocities. Along the ΓK′ direction we obtain *λ*_F_ = 0.53 (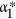), 0.48 (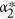), 0.30 (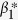 and 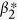) for the π^*^ bands, and *λ*_F_ = 0.68 for the IL band. Along the other two directions ΓM′ and M′K′ the mass enhancement parameters are up to a factor of three smaller than the corresponding values along ΓK′, suggesting a rather anisotropic EPC. Our findings are in agreement with ARPES studies on bulk KC_6_ and CaC_6_ superconductors^10^,^22^ and also with recent work on Rb-intercalated bilayer graphene^3^.

We now move from the normal state to the superconducting state, and consider an electron-phonon pairing mechanism in analogy with bulk CaC_6_. Figure 2(a) shows a comparison between the isotropic Eliashberg spectral functions, *α*^2^*F*(*ω*), and the cumulative total EPC, *λ*(*ω*), calculated for C_6_CaC_6_ and for bulk CaC_6_. In both cases we can distinguish three main contributions to the EPC, to be associated with the Ca_*xy*_ vibrations (~10 meV), the out-of-plane C_*z*_ vibrations (~70 meV), and the in-plane C_*xy*_ modes (~180 meV). The Eliashberg functions of C_6_CaC_6_ and CaC_6_ look similar in shape, and the total EPC is *λ* = 0.71 in both cases. However the relative contributions of each set of modes differ considerably. The low-energy Ca modes are slightly softer in C_6_CaC_6_, leading to a larger contribution to the EPC than in CaC_6_. These modes account for 60% of the total coupling in the bilayer, while they only account for less than 30% of the coupling in bulk CaC_6_. In both cases the EPC strength associated with the in-plane C-C stretching modes is too weak (15% of the total) to make a sizable contribution to the superconducting pairing. This is somewhat counterintuitive, given that the C_*xy*_ modes lead to the most pronounced kinks in the spectral function in Fig. 1.

The smaller contribution of the out-of plane C_*z*_ modes to the EPC and the softening of the in-plane Ca_*xy*_ vibrations, obtained when going from the bulk to the bilayer, are similar to the results found for Li- and Ca-decorated monolayer graphene^29^. In the monolayer case, the removal of quantum confinement causes a shift of the IL wave function farther away from the graphene layer as compared to bulk, and, therefore, the EPC coupling between π^*^ and IL states mediated by C_*z*_ vibrations is reduced. Although in the bilayer case the IL state is strongly localized around the Ca atom, the fact that the interlayer charge density is only present between the graphene layers gives rise to a weaker coupling of the out-of-plane C_*z*_ modes with the interlayer electrons, and, therefore, a lower contribution to the global EPC.

To check whether the softening of the low energy Ca_*xy*_ modes is due to the difference in the structural parameters between the bilayer and the bulk, we recalculate the vibrational spectrum of bilayer C_6_CaC_6_ after setting the in-plane lattice constant and the interlayer distance to the values of the bulk. As shown in Supplementary Fig. S3, the in-plane C_*xy*_ modes (*ω* > 105 meV) soften due to the increase in the in-plane lattice constant, and the out-of-plane C_*z*_ and Ca_*z*_ modes (20 < *ω* < 45 meV) harden due to the decrease in the interlayer distance. On the other hand, the two lowest-lying modes involving mainly in-plane Ca_*xy*_ vibrations are considerably less affected, although a closer inspection reveals an overall slight hardening of approximately 1 meV, particularly along the ΓM′ and M′K′ directions. This suggests that the observed softening of the low-energy Ca_*xy*_ modes from bulk to bilayer is most likely caused by changes in the electronic structure which is consistent with an increased density of states at the Fermi level.

In order to determine the superconducting critical temperature of C_6_CaC_6_ we solve the anisotropic Eliashberg equations^26^,^30^,^31^, with the Coulomb pseudopotential μ^*^ calculated from first principles (the calculation of μ^*^ is discussed below). In Fig. 2(b,c) we plot the energy-dependent distribution of the superconducting gap, separated into contributions corresponding to the two sets of Fermi surfaces centered around the Γ and K′ points. We see that two distinct gaps open on the Γ-centered electron pockets, with average values Δ_1_ = 0.55 meV and Δ_2_ = 1.29 meV at zero temperature. The Δ_1_ gap is characterized by a very narrow energy profile and the EPC on these pockets is essentially isotropic, resulting primarily from the coupling with the Ca_*xy*_ phonons [Fig. 2(d)]. The Δ_2_ gap exhibits a much broader energy profile (0.82 < Δ_2_ < 1.75 meV) and originates mainly from the coupling to Ca modes and out-of-plane C_*z*_ phonons [Fig. 2(e)]. In between the two Γ-centered gaps, a third gap with an average value Δ_3_ = 0.99 meV opens on the triangular hole pockets around K′ [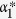 states] [Fig. 2(f)]. These states couple primarily to C_*xy*_ phonons and the gap has an anisotropic character with a large spread in energy (0.73 < Δ_3_ < 1.25 meV). In Supplementary Fig. S4 we show the anisotropic EPC parameters *λ*_*k*_ leading to this superconducting gap structure. For completeness, we compare our results with the gap structure of bulk CaC_6_. In the latter case, only one superconducting gap is predicted (see Supplementary Fig. S5), in agreement with previous theoretical studies^20^,^32^ and experiments^33^,^34^. Although multiple sheets of the Fermi surface contribute to the superconducting gap, there is no separation into distinct gaps, giving rise to a smeared multigap structure^20^,^32^. This situation is similar to the Δ_2_ and Δ_3_ gaps in the bilayer case. Based on our results we suggest that in C_6_CaC_6_ high-resolution ARPES experiments might be able to resolve two distinct gaps on the electron pockets, corresponding to Δ_1_ and Δ_2_, but only one gap on the triangular hole pockets, corresponding to Δ_3_.

The calculations of the gap function and the superconducting critical temperature in Fig. 2(b,c) require the knowledge of the Coulomb pseudopotential μ^*^. In order to determine this parameter we first calculate the dimensionless electron-electron interaction strength within the random-phase approximation, obtaining *μ* = 0.254 (see Methods). Then we renormalize this interaction using μ^*^ = *μ*/[1 + *μ*log(*ω*_pl_/*ω*_ph_)]^35^, where *ω*_pl_ and *ω*_ph_ are characteristic electron and phonon energy scales, respectively. We set *ω*_pl_ = 2.5 eV, corresponding to the lowest plasmon resonance in GICs^36^,^37^, and *ω*_ph_ = 200 meV, corresponding to the highest phonon energy in C_6_CaC_6_. Figure 2(g) shows the variation of the Coulomb pseudopotential *μ*_k_ across the Fermi surface. The repulsive interaction is strongest on the electron pockets, and weakest on the hole pockets. Since the EPC exhibits a very similar anisotropy, the net coupling strength 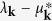 is only moderately anisotropic and can be replaced by a single, average μ^*^ in the Eliashberg equations. From Fig. 2(g) we obtain μ^*^ = 0.155, and this is the value employed in Fig. 2(b,c). As we can see in Fig. 2(b,c), our Eliashberg calculations yield a superconducting critical temperature *T*_c_ = 8.1 K, which is only slightly higher than the experimental value 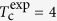 K reported in ref.^4^.

In order to check the role of anisotropic Coulomb interactions we repeat the calculations by considering a Coulomb pseudopotential resolved over the electron and hole pockets. In this case we find the decrease in *T*_c_ to be very small, Δ*T*_c_ = 0.3 K. Furthermore we explore the sensitivity of the calculated *T*_c_ to the choice of the characteristic phonon energy *ω*_ph_. To this aim we solve the Eliashberg equations again, this time by setting *ω*_ph_ equal to the Matsubara frequency cutoff (5 × 200 meV, see Methods). This alternative choice leads to μ^*^ = 0.207 and *T*_c_ = 6.8 K (Supplementary Fig. S6), which is in even better agreement with experiment. For completeness in Supplementary Fig. S7 we also show the dependence of *T*_c_ on the characteristic energy *ω*_c_ as obtained using the standard McMillan formula^38^. Consistent with our Eliashberg calculations, we find that large variations of *ω*_c_ only change *T*_c_ by a few K’s. These additional tests show that our results are solid, therefore we can safely claim that the *ab initio* Eliashberg theory yields *T*_c_ = 6.8–8.1 K for C_6_CaC_6_. The close agreement between these values and experiment supports the notion that Ca-intercalated bilayer graphene is a conventional phonon-mediated superconductor.

In conclusion, we studied entirely from first principles the electron-phonon interaction and the possibility of phonon-mediated pairing in the newly-discovered superconducting C_6_CaC_6_. We showed that the Ca vibrations play an important role in the pairing but do not carry a sharp signature in the normal-state band structure; conversely the high-frequency in-plane C*xy* vibrations lead to pronounced photoemission kinks but have a small contribution to the pairing. The good agreement between the critical temperature calculated here and the recent experiments of ref. 4 indicate that Ca-intercalated bilayer graphene is an electron-phonon superconductor. The present work calls for high-resolution spectroscopic investigations, as well as for calculations based on alternative *ab initio* methods^39^,^40^, in order to test our prediction of two distinct superconducting gaps in C_6_CaC_6_.

## Methods

The calculations are performed within the local density approximation to density-functional theory^41^,^42^ using planewaves and norm-conserving pseudopotentials^43^,^44^, as implemented in the Quantum-ESPRESSO suite^45^. The planewaves kinetic energy cutoff is 60 Ry and the structural optimization is performed using a threshold of 10 meV/Å for the forces. C_6_CaC_6_ is described using the 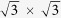 *R*30° supercell of graphene with one Ca atom per unit cell, and periodic images are separated by 15 Å. The optimized in-plane lattice constant and interlayer separation are *a* = 4.24 Å and *d* = 4.50 Å, respectively. Bulk CaC_6_ is described using the rhombohedral lattice, and the optimized lattice constant and rhombohedral angle are *a* = 5.04 Å and *α* = 50.23°, respectively. The electronic charge density is calculated using an unshifted Brillouin zone mesh with 24^2^ and 8^3^ k-points for C_6_CaC_6_ and CaC_6_, respectively, and a Methfessel-Paxton smearing of 0.02 Ry. The dynamical matrices and the linear variation of the self-consistent potential are calculated within density-functional perturbation theory^46^ on the irreducible set of regular 6^2^ (C_6_CaC_6_) and 4^3^ (CaC_6_) **q**-point grids.

The electron self-energy, spectral function, and superconducting gap are evaluated using the EPW code^25^,^26^,^47^. The electronic wavefunctions required for the Wannier-Fourier interpolation^48^,^49^ in EPW are calculated on uniform unshifted Brillouin-zone grids of size 12^2^ (C_6_CaC_6_) and 8^3^ (CaC_6_). The normal-state self-energy is calculated on fine meshes consisting of 100,000 inequivalent **q**-points, using a broadening parameter of 10 meV and a temperature *T* = 20 K. For the anisotropic Eliashberg equations we use 120 × 120 (40 × 40 × 40) k-point grids and 60 × 60 (20 × 20 × 20) **q**-point grids for C_6_CaC_6_ (CaC_6_). The Matsubara frequency cutoff is set to five times the largest phonon frequency (5 × 200 meV), and the Dirac delta functions are replaced by Lorentzians of widths 100 meV and 0.5 meV for electrons and phonons, respectively. The technical details of the Eliashberg calculations are described extensively in ref. 26.

The electron-electron interaction strength is obtained as *μ* = *N*_F_〈〈*V*_k,k**′**_〉〉_F*S*_, where *N*_F_ is the density of states at the Fermi energy, 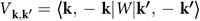, and *W* is the screened Coulomb interaction within the random phase approximation^50^. Here 〈〈⋅〉〉_F*S*_ indicates a double Fermi-surface average, and k stands for both momentum and band index. The screened Coulomb interaction is calculated using the Sternheimer approach^51^,^52^. Linear-response equations are solved using 26 × 26 Brillouin-zone grids, corresponding to 70 inequivalent points. The energy cutoff of the dielectric matrix is 10 Ry (815 planewaves).

## Additional Information

**How to cite this article**: Margine, E. R. *et al.* Electron-phonon interaction and pairing mechanism in superconducting Ca-intercalated bilayer graphene. *Sci. Rep.* **6**, 21414; doi: 10.1038/srep21414 (2016).

## Supplementary Material

Supplementary Information

## Figures and Tables

**Figure 1:**
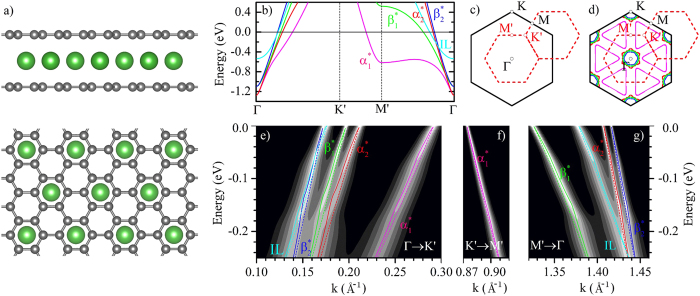
Crystal structure, band dispersion, Fermi surface, and spectral function of bilayer C_6_CaC_6_. (**a**) Side- and top-view of a ball-and-stick model of C_6_CaC_6_, with C in grey and Ca in green. The structure is analogous to bulk CaC_6_^53^. (**b**) Band structure of C_6_CaC_6_. The outermost π^*^ bands (with respect to Γ point) are labeled as 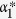 (magenta line) and 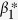 (green line), the innermost π^*^ bands are labeled as 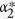 (red line) and 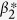 (blue lines). The interlayer band is labeled as IL (cyan line). (**c**) Brillouin zones of a graphene unit cell (black full lines) and a 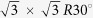 graphene supercell (red dashed lines). (**d**) Two-dimensional Fermi surface sheets of C_6_CaC_6_, with the same color code as in (**b**). (**e**–**g**) Calculated spectral function of bilayer C_6_CaC_6_ in the normal state, along the same high-symmetry directions as shown in (**b**). The band structures with (solid lines) or without EPC (dashed lines) are overlaid on top of the spectral function.

**Figure 2:**
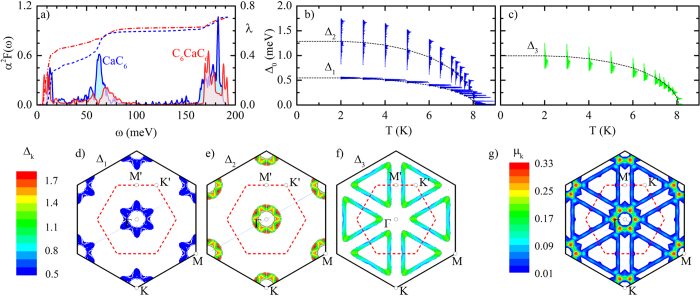
Electron-phonon coupling and superconducting gap function of bilayer C_6_CaC_6_. (**a**) Eliashberg spectral function and cumulative EPC calculated for CaC_6_ (blue) and C_6_CaC_6_ (red). The solid lines are for *α*^2^*F*(*ω*) (left scale), the dashed lines are for *λ*(*ω*) (right scale). (**b**–**c**) Energy distribution of the anisotropic superconducting gaps Δ_k_ of C_6_CaC_6_, centered around the Γ and K′ points as a function of temperature. The gaps were calculates using the *ab initio* Coulomb pseudopotential μ^*^ = 0.155. The dashed black lines indicate the average values of the gaps. The gaps vanish at the critical temperature *T*_c_ = 8.1 K. The color-coded gaps at the lowest temperature refer to the segments Δ_1_, Δ_2_, and Δ_3_ discussed in the text, and can approximately be identified with the panels (**d**–**f**), respectively. (**d**–**f**) Momentum-resolved superconducting gap Δ_*k*_ (in meV) on the Fermi surface at zero temperature: (**d**,**e**) correspond to the lower gap Δ_1_ and the upper gap Δ_2_ centered around the Γ point, (**f**) corresponds to the Δ_3_ centered around the K′ point. (**g**) Dimensionless anisotropic Coulomb pseudopotential *μ*_k_ on the Fermi surface. For clarity in (**d**–**g**) the values correspond to electrons within ±250 meV from the Fermi energy (hence the ‘thick’ Fermi surface sheets).
