# Supplementary material for: Electron-phonon interaction and pairing mechanism in superconducting Ca-intercalated bilayer graphene
Source: Sci Rep. 2016 Feb 19;6:21414. doi: 10.1038/srep21414 (PMC4759825; doi:10.1038/srep21414)
Supplement: Supplementary Information [file srep21414-s1.pdf]

Electron-phonon interaction and pairing mechanism in superconducting Ca-intercalated bilayer graphene

E. R. Margine[1,*], Henry Lambert[2], and Feliciano Giustino[2]

[1]Department of Physics, Applied Physics and Astronomy, Binghamton University, State University of New York, PO Box 6000, Binghamton, New York 13902-6000, USA
[2]Department of Materials, University of Oxford, Parks Road, Oxford OX1 3PH, United Kingdom
[*]rmargine@binghamton.edu

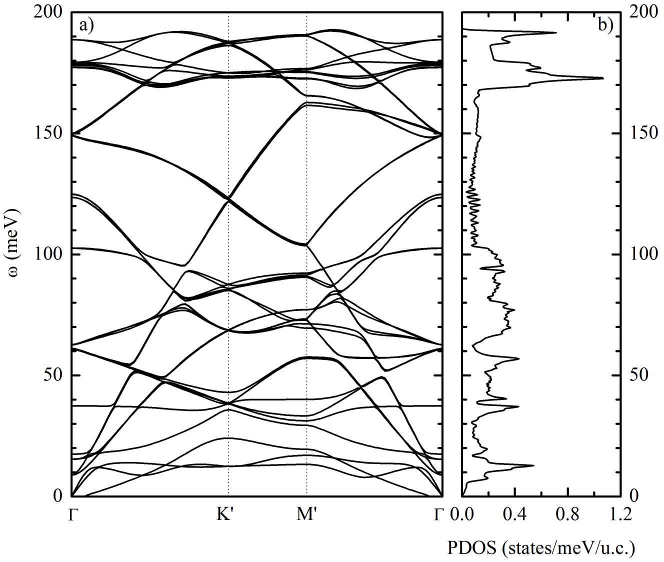

Supplementary Figure 1. Calculated phonon dispersion and phonon density of states for C_6CaC_6. (a) phonon dispersion relations and (b) phonon density of states.

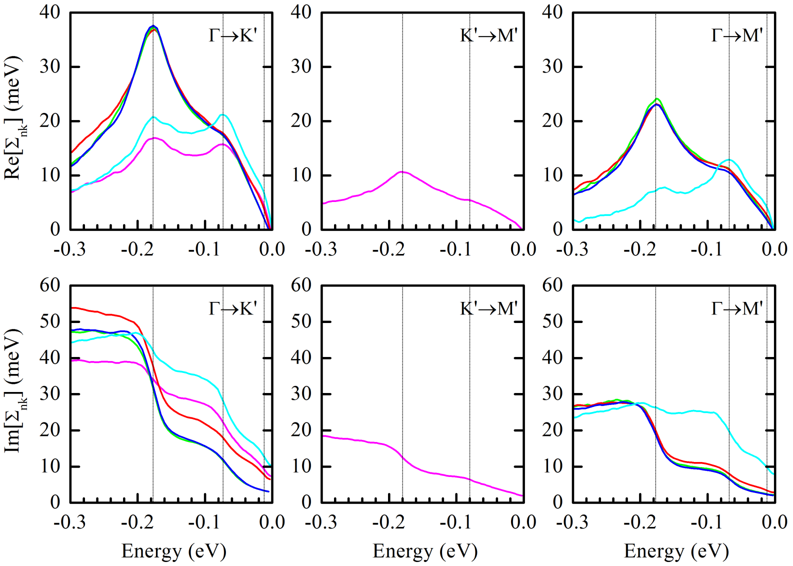

Supplementary Figure 2. Calculated electron-self energy for C_6CaC_6. Real part (top row) and imaginary part (bottom row) of the electron self-energy Σ arising from the electron-phonon interaction, calculated for Ca-intercalated bilayer graphene in the normal state. The color code is the same as in Fig. 1(b) of the main text: for the π^* bands magenta is for α_1^*, green for β_1^*, red for α_2^*, and blue for β_2^*. The interlayer band is in cyan. The high-symmetry directions in the Brillouin zone are indicated in each panel and correspond to Fig. 1(c) of the main text. The vertical grey lines indicate the vibrational frequencies of the three prominent features at 12 meV, 70 meV, and 180 meV discussed in the main text.

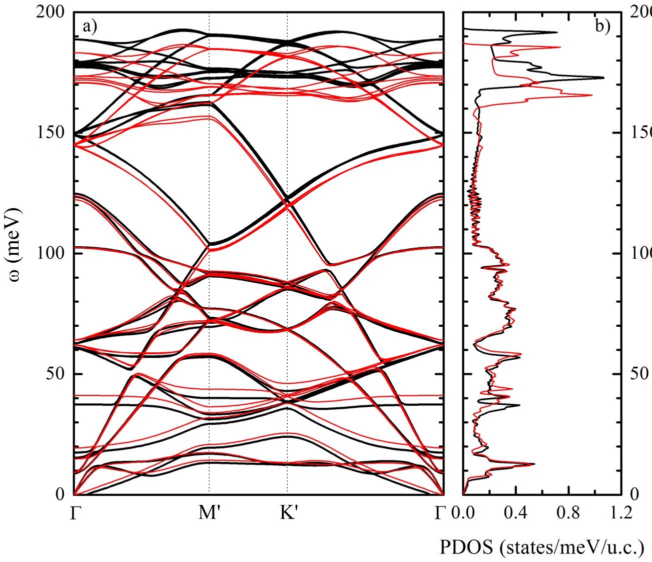

Supplementary Figure 3. Calculated phonon dispersion and phonon density of states for C_6CaC_6. (a) phonon dispersion relations and (b) phonon density of states. Black lines correspond to the relaxed structural parameters of bilayer C_6CaC_6 ($a = 4.2429$ Å and $c = 4.5027$ Å) and red lines correspond to the relaxed structural parameters of bulk CaC_6 ($a = 4.2784$ Å and $c = 4.3937$ Å).

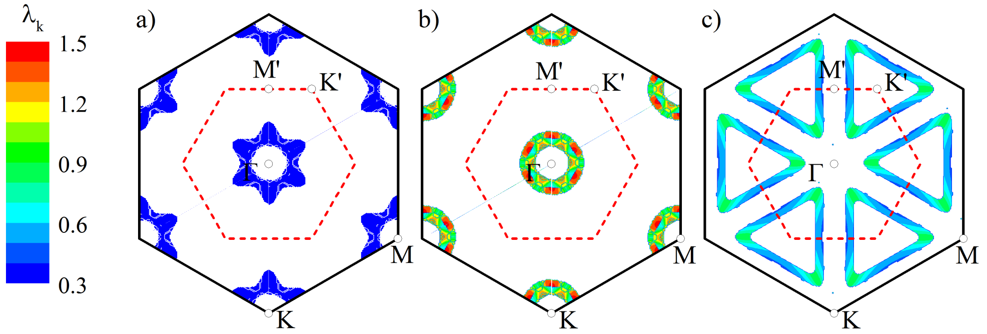

Supplementary Figure 4. Calculated momentum-resolved electron-phonon coupling parameters $\lambda_{\mathbf{k}}$ on the Fermi surface of Ca-intercalated bilayer graphene. These are the EPC values employed in the solution of the anisotropic Eliashberg equations leading to Figs. 2(d)-(f) in the main text. As in the main text, the data points correspond to electrons within ±250 meV from the Fermi energy (this explains why we have 'thick' Fermi surface sheets as opposed to thin lines).

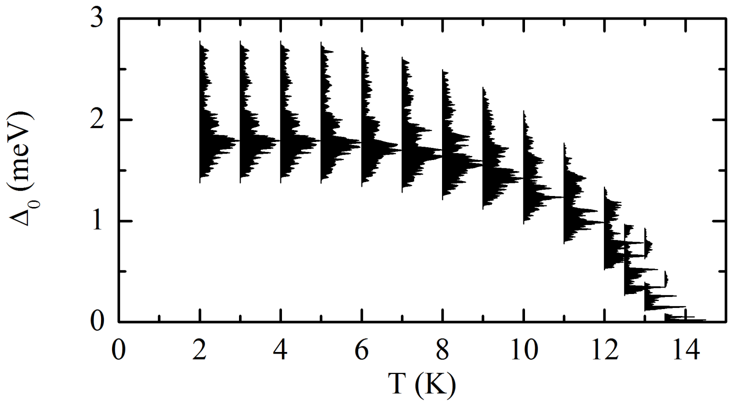

Supplementary Figure 5. Distribution of the superconducting gap function calculated for bulk CaC_6. This is to be compared with Figs. 2(b)-(c) of the main text. Unlike C_6CaC_6, in this case we do not find two distinct superconducting gaps. In this case the Eliashberg equations were solved using $\mu^* = 0.14$, as calculated within the random-phase approximation (see Methods in the main text). The calculated critical temperature $T_c = 13.5$ K is in good agreement with the measured value $T_c^{\text{exp}} = 11.5$ K (Ref. [11] of the main text).

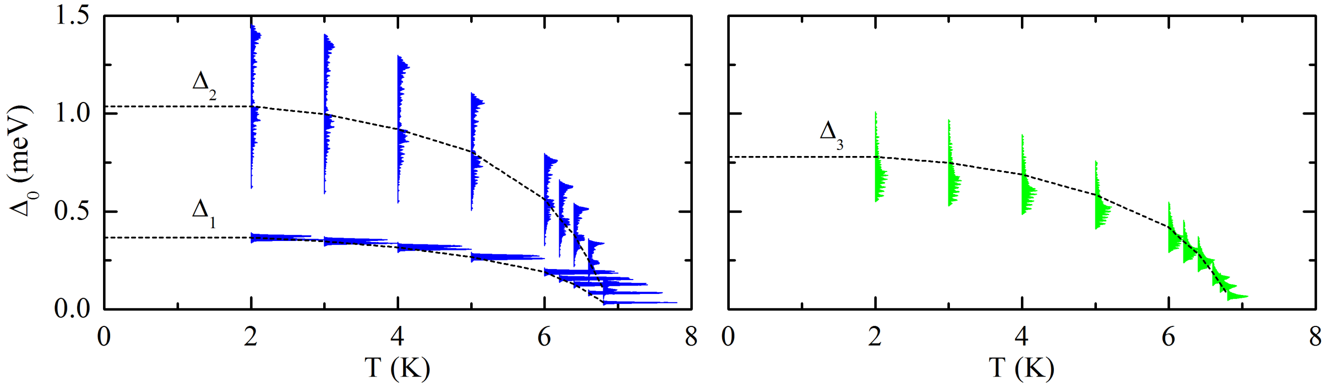

Supplementary Figure 6. **Distribution of the superconducting gap function calculated for Ca-intercalated bilayer graphene.** The computational setup is the same as in Figs. 2(b)-(c) of the main text, except in this case we set the characteristic phonon energy ω_{ph} to the Matsubara frequency cutoff (5×200 meV), leading to $\mu^* = 0.207$. In this case the calculated critical temperature is $T_c = 6.8$ K. The dashed lines are guides to the eye.

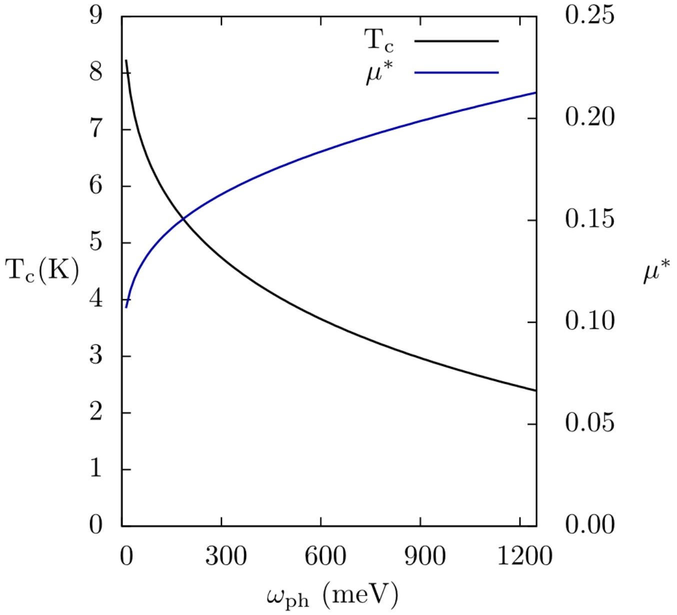

Supplementary Figure 7. **Dependence of T_c and μ^* on the characteristic phonon energy ω_c.** Sensitivity of the calculated critical temperature of C_6CaC_6 to the choice of the characteristic phonon energy ω_c in $\mu^* = \mu/[1+\mu \log(\omega_{\text{pl}}/\omega_{\text{ph}})]$. For simplicity we plot the critical temperature obtained by using the McMillan equation $T_c = \omega_{\log}/1.2 \exp\{-1.04(1+\lambda)/[\lambda - \mu^*(1+0.62\lambda)]\}$, with $\omega_{\log} = 21.1$ meV, $\lambda = 0.71$, and $\mu = 0.254$. The variation of T_c between $\omega_c = 200$ meV (the highest phonon energy) and $\omega_c = 5 \times 200$ meV (the Matsubara frequency cutoff) is $\Delta T_c = 2.6$ K, in good agreement with our *ab initio* Eliashberg calculations described in the main text (for which $\Delta T_c = 1.6$ K).
